# The genetic analysis of eight families with hemophilia B in Mongolia: Identification of two novel mutation

**DOI:** 10.1002/mgg3.2495

**Published:** 2024-09-13

**Authors:** Purevdorj Munkhuu, Munkhtsetseg Bazarragchaa, Purevdorj Ichinkhorloo, Ki‐Young Yoo, Enkh‐Amar Ayush, Ochbadrakh Batjargal, Erdenebayar Namjil, Sarantuya Jav, Erkhembulgan Purevdorj, Sodnomtsogt Lkhagvasuren

**Affiliations:** ^1^ Department of Molecular Biology and Genetics, School of Bio‐Medicine Mongolian National University of Medical Science Ulaanbaatar Mongolia; ^2^ Hematology Korean Hemophilia Foundation Seoul South Korea; ^3^ Department of Gastroenterology, School of Medicine Mongolian National University of Medical Science Ulaanbaatar Mongolia; ^4^ National Center for Transfusion Medicine Peace Avenue Ulaanbaatar Mongolia; ^5^ Division for Science and Technology Mongolian National University of Medical Sciences Ulaanbaatar Mongolia

**Keywords:** carrier, F9, hemophilia B, Mongolia

## Abstract

**Background:**

This study aimed to conduct molecular diagnostics among individuals with hemophilia B (HB) and carriers of hemophilia in Mongolia.

**Methods:**

Eight patients (six severe, two mild) with HB and their 12 female relatives were enrolled from eight families. Sanger sequence was performed for mutation identification. The questionnaire survey was conducted to evaluate carrier symptoms in female relatives.

**Results:**

Two families had a history of HB. A total of five different variants (c.223C > T; c.344A > G; c.464G > C; c.187_188del; and c.1314_1314delA) were identified in six patients with severe HB. Of these, two (c.187_188del and c.1314_1314delA) were novel. No variant in the entire *F9* was found in two patients with mild HB. Nonsense c.223C > T (p.Arg75*) mutation was detected in two unrelated patients. Carrier testing identified five mothers as carriers, while one younger sister was a non‐carrier. The carrier status of six female relatives of the two mild patients remained undetermined. By questionnaire survey, only one of the five genetically identified carriers displayed noticeable symptoms of being a carrier.

**Conclusion:**

The novel variants c.187_188del and c.1314_1314delA can cause severe hemophilia B. This study did not observe a significant association between symptoms and carrier status in the five carriers.

## Graphical abstract

Variants in F9 gene were found in six out of eight families. Two variants were novel or not registered previously. Only one female relative displayed indicative symptoms of hemophilia carrier.

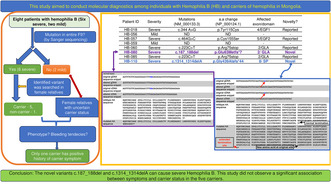

## 1 INTRODUCTION

Hemophilia B (HB, OMIM #306900; ICDX: D67) is an X‐linked recessive bleeding disorder caused by various mutations in the coagulation factor IX gene (*F9*, MIM #300746), resulting in a qualitative or quantitative deficiency of coagulation factor IX (FIX) (Shen et al., 2022). Earlier (in 1952), HB was also named “Christmas disease” due to the first patient named Stephen Christmas (Biggs et al., 1952). The *F9* consists of eight exons, 33 kb long, located on Xq27.1 and generates a 2802 bp mRNA that encodes a protein with 461 amino acid residues, containing five domains (GLA, EGF1, EGF2, Act, and serine protease) (Gomez & Chowdary, 2014; Shen et al., 2022). According to Mendelian inheritance, males are affected, and females become asymptomatic carriers (Bolton‐Maggs & Pasi, 2003). Coagulation Factor IX plays a crucial role in the intrinsic pathway of the coagulation system. Activated FIX and its cofactor Factor VIII activate Factor X, which converts prothrombin to thrombin. Activated thrombin converts fibrinogen to fibrin, forming the clot during secondary hemostasis (Brummel Ziedins & Mann, 2014). The phenotype of HB is categorized as severe (FIX level: less than 1%), moderate (FIX level: 1–5%), and mild (FIX level: 5%–40%) forms, all based on the baseline level of FIX in plasma (Peyvandi et al., 2016; Santagostino & Fasulo, 2013). HB occurs one in 30,000 male births; clinical symptoms of hemophilia are characterized by easy bruises, bleeding into the joints (hemarthrosis), nosebleeds (epistaxis), intramuscular hematoma (pseudotumor), prolonged bleeding after wounds or injuries, and life‐threatening bleeds (bleeds into the central nervous system) in an untreated patient (Srivastava et al., 2020). As of recently, 1083, 1094, 1244, and 1283 mutations in the *F9* were registered in The CDC hemophilia B mutation project (Li et al., 2013), the Human *F9* gene variant database (University College London, 2021), The European Association for Hemophilia and Allied Disorders (McVey et al., 2020), and Human Genome Mutation Database 2022), respectively. Point mutations constitute the majority of *F9* mutations, accounting for approximately 65–76.7% of the total (Goodeve, 2015; Miller, 2021; Rallapalli et al., 2013). Utilizing a combination of exon PCR, Sequence analysis, and Multiplex Ligation‐dependent Probe Amplification (MLPA), a 97% mutation detection rate can be achieved (Goodeve, 2015; Konkle et al., 2017). Detecting the causative mutation in *F9* is crucial for establishing a mutation profile in the affected families, detecting hemophilia carriers, and enabling prenatal diagnosis (Goodeve, 2015). Carrier women with heterozygotic genotypes typically present as asymptomatic. However, those with 0.05–0.60 IU/mL of Factor IX are at increased risk of bleeding after the tooth extraction, postpartum hemorrhage, and surgery than non‐carrier women (Plug et al., 2006). A significantly higher risk of prolonged bleeding following tooth extraction and surgical procedures was observed in carrier women compared to non‐carrier women, with relative risks of 2.3 and 2.4, respectively (Mauser‐Bunschoten, 2008). The ability to identify heterozygote carriers of HB was limited prior to the availability of gene sequencing (Miller, 2021). Genetic testing is a vital tool in identifying carriers and enabling prenatal diagnosis. It is recommended that potential carriers who are sisters and aunts of hemophilia carriers undergo formal genetic testing when they are mature enough to understand the diagnostic implications (Dunn et al., 2008; Srivastava et al., 2020). This is the first report of a molecular genetic study of HB in Mongolia.

## 2 MATERIALS AND METHODS

### 2.1 Ethical compliance

According to the Declaration of Helsinki, informed consent was obtained from each patient or their legal guardians. This study was previously approved by the Bio‐Ethical Committee of the Mongolian National University of Medical Sciences (Approval no. 12‐13/11) and conducted in accordance with the Principles of the Declaration of Helsinki.

### 2.2 Subjects

Eight male patients with HB (aged 2 to 33) and their 12 female relatives (aged 11 to 42) from eight families participated in this study. The patients had already been diagnosed and were receiving healthcare services at two different tertiary‐level hospitals in Mongolia (National Center for Maternal and Child Health and The State First Central Hospital). The disease severity was determined based on baseline FIX levels and the diagnosis provided by Hemophilia Treatment Centers. Additional data (severity, diagnosis, age onset, and type of hemophilia) was collected and reviewed from the patient card. A pedigree graph with three generations was drawn for each of the probands. Bleeding tendencies suggestive of carrier status were obtained from female relatives through a questionnaire. The survey collected specific information about prolonged bleeding after surgery or trauma, easy bruising, duration of menstruation, and postpartum hemorrhage.

### 2.3 Sample collection and DNA extraction

A peripheral blood sample (3–7 mL) was collected in an EDTA tube, followed by the isolation of the buffy coat. The genomic DNA was then extracted from the buffy coat using the Qiagen DNA blood mini kit (Cat No.: 51106, Qiagen Inc., Germany). Subsequently, the isolated DNA samples were stored at −20°C until the commencement of the test.

### 2.4 Molecular genetic analysis

Eight primer pairs were used to detect variants in an entire exon and exon‐intron boundaries of *F9*. The polymerase chain reaction (PCR) and sequence test primers are shown in Table S1 in Supplementary Material. The exon PCR of *F9* was performed using the AmpliTaq Gold PCR mix (Cat No.: 4311806), followed by purification of the PCR products using the Qiaquick PCR kit (Cat No.: 28104). Subsequently, a sequencing PCR was carried out using the Big dye® terminator v3.1 cycle sequencing kit (Cat No.: 4337455) according to the manufacturer's instructions, followed by purification of the PCR products using the Qiaquick PCR kit (Cat no: 28104). The Sequence PCR products were mixed with 10 mL of Hi‐Di formamide and subsequently incubated at 98°C for 3 minutes prior to being subjected to Sanger sequencing using the ABI genetic analyzer (Foster City, California, USA).

### 2.5 *F9* sequencing analysis

Sequence raw data was aligned with the reference sequence of *F9* (Gene Bank accession number: NG_007994.1, mRNA coding sequence: NM_000133.3) using the software Sequencher 5.3 and Codon code aligner v10.0.02. Variants found in patients were investigated in female relatives to determine carrier status. Variants were numbered according to the Human Genome Variation Society (HGVS) guidelines, using the Adenine of the initiator ATG codon as nucleotide +1 (den Dunnen et al., 2016; Human Genome Mutation Society, 2020). The variants detected in *F9* were looked at with three different online sources to verify if they were previously reported: 1st: CDC Hemophilia Mutation Project (CHBMP: https://www.cdc.gov/ncbddd/hemophilia/champs.html); 2nd Human Genome Mutation Society (2020) (https://www.hgmd.cf.ac.uk/ac/gene.php?gene=F9), 3rd Human *F9* gene variant database (https://www.factorix.org/index.php).

### 2.6 Bio‐informatic computational (in Silico) analysis of the variant effect

The effect of each variant was analyzed using most common four distinct online tools: 1st SIFT (Score: “Tolerent >0.05,” “intolerant <0.05 is deleterious or affect protein function”), 2nd Mutation taster (Grantham Matrix score: 0–215), 3rd Polyphen 2 program (score ranged from 0 to 1, “0 is benign”, “1 is probably damaging”) and 4th FATHMM (Tolerated and Damaging). Novel variants were documented and confirmed in the Leiden Open Variation Database (LOVD) system (accessible at https://databases.lovd.nl/shared/variants). The pathogenicity of variants was interpreted following ACMG guidelines (Richards et al., 2015).

## 3 RESULTS

Table 1 shows the general information (ID, age, severity, family history) of eight male patients and their 12 female relatives, as well as variants found in members of eight families. The pedigree analysis revealed that the two families had a history of HB in previous generations or among relatives (Table 1).

**TABLE 1:** The general information of study participants.

Family number	Family history	Members	ID	Age	Severity^a^/IX activity	Identified variants (NM_000133.3)
Family‐1	Negative	Proband	HB‐018	33	Severe/<1%	c.344 A>G
		Y‐sister	HB‐012	19	90%	Non‐carrier
Family‐2	Negative^b^	Proband	HB‐056	4	Mild/16%	ND
		Mother	HB‐065	38	NA	
		Aunt	HB‐066	36	NA	
		Aunt	HB‐067	34	NA	
		Aunt	HB‐068	20	NA	
Family‐3	Negative	Proband	HB‐057	4	Severe/<1%	c.464G>C
		Mother	HB‐072	28	NA	Carrier
Family‐4	Negative	Proband	HB‐059	18	Mild/15%	ND
		Mother	HB‐081	42	NA	
		Y‐sister	HB‐082	11	NA	
Family‐5	Positive	Proband	HB‐060	3	Severe/<1%	c.223C>T
		Mother	HB‐075	28	NA	Carrier
Family‐6	Positive	Proband	HB‐080	4	Severe/<1%	c.187_188del
		Mother	HB‐071	24	NA	Carrier
Family‐7	Negative	Proband	HB‐085	4	Severe/<1%	c.223C>T
		Mother	HB‐088	30	NA	Carrier
Family‐8	Negative	Proband	HB‐110	2	Severe/<1%	c.1314_1314delA
		Mother	HB‐108	22	30%	Carrier

Abbreviations: FIX, activity level was tested in only patients; Y‐sister, younger sister.

^a^ Severity was based on baseline FIX level (severe: FIX<1%; moderate: 1–5%; mild: 6%–40%).

^b^ A female relative not participated has a history of recurrent epistaxis and joint pain; ND, not determined.

Five distinct variants, including two missenses, one nonsense, and two novel deletions, were found in six out of eight families. All the identified variants and their molecular genetic information have been listed in Table 2. Moreover, no mutation was detected in two patients with mild HB. Two unrelated patients (HB‐060 and HB‐085) had the same nonsense (c.223C>T) mutation. Two novel deletions (c.187_188del and c.1314_1314delA) were found in two patients with severe HB, respectively (see Table 2). Sequence chromatogram figures of the identified single point variants in patients and their relatives have been shown in Figures S1–S3 in Supplementary Materials.

**TABLE 2:** The variants found in the *F9* gene.

Patient ID	Severity	Mutations (NM_000133.3)	A.A change^b^ (NP_000124.1)	Affected exon/domain	Novelty?
HB‐018	Severe	c.344 A>G	p.Tyr115Cys	4/EGF1	Reported
HB‐056	Mild	ND	ND	‐	‐
HB‐057	Severe	c.464G>C	p.Cys155Ser	5/EGF2	Reported
HB‐059	Mild	ND	ND	‐	‐
HB‐060	Severe	c.223C>T	p.Arg75stop	2/GLA	Reported
HB‐080	Severe	c.187_188del^a^	p.Glu63Metfs*7	2/ GLA	Novel
HB‐085	Severe	c.223C>T	p.Arg75stop	2/GLA	Reported
HB‐110	Severe	c.1314_1314delA^a^	p.Gly439Alafs*44	8/SP	Novel

Abbreviation: ND, not detected; Ref, reference; SP, serine protease.

^a^ Variant were reported to the LOVD (https://databases.lovd.nl/shared/genes/F9).

^b^ Amino acids numbering followed HGVS nomenclature.

**FIGURE 1:**
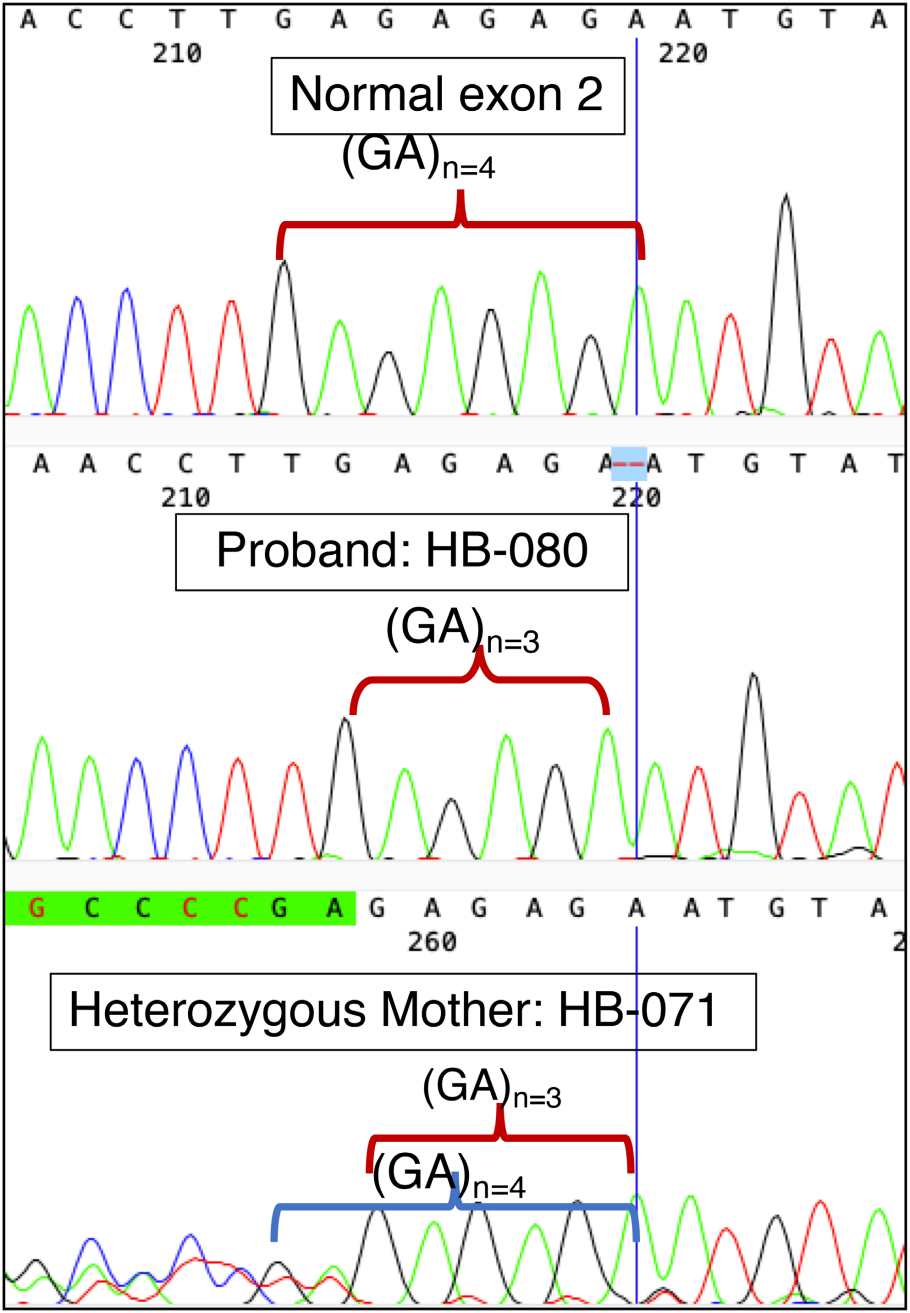
Sequence chromatogram of the novel c.187_188del variant. The reference sequence (NG_007994.1) of F9 is shown with at the top. “Normal exon 2” represents the wild‐type sequence. HB‐080 is a patient with the variant. The patient's mother (HB‐071) is the carrier with a heterozygote genotype.

**FIGURE 2:**
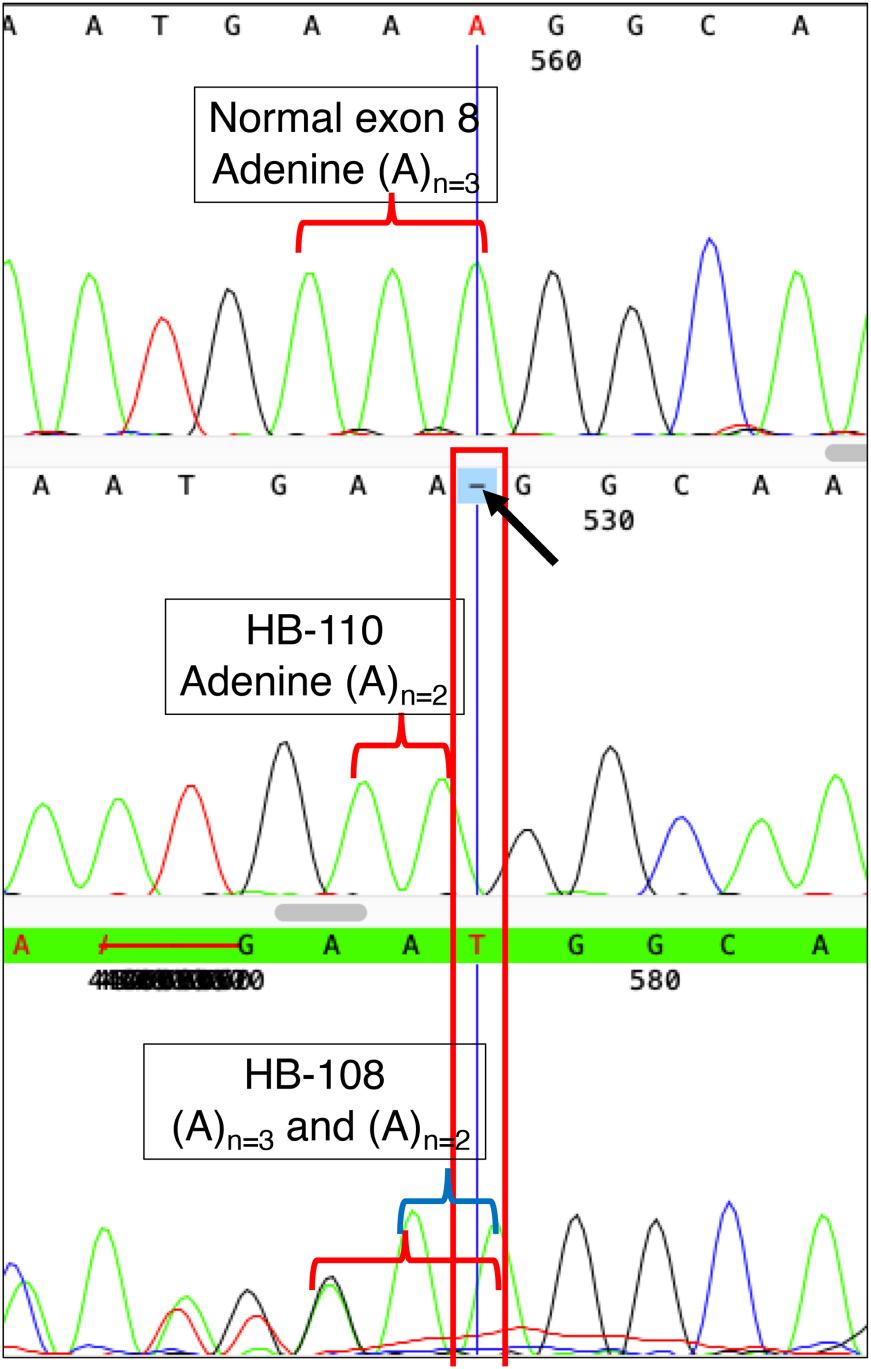
Sequence chromatogram of novel c.1314_1314del variant. One normal sequences (wild type 1, 2, 3) of exon 8 were compared to the sequences of the patient (HB‐110) and his mother (HB‐108). The red line indicates the altered position of the nucleotide, while the black arrow highlights the missing base in the patient's sequence.

These novel variants have been registered as publicly in the LOVD system (available on: https://databases.lovd.nl/shared/variants/F9/unique?search_var_status=%3D%22Marked%22%7C%3D%22Public%22). Figures 1 and 2 show the sequence alignment of the Novel c.187_188del and 1314_1314del variants, respectively (see Figures 1 and 2).

The bioinformatics tools predict that all the identified variants are potentially damaging or causative for HB (see Table 3).

**TABLE 3:** Prediction of variant effect by bioinformatics software.

A.Acid change^b^ (variant)	Affected domain	Mutation taster	Polyphen2	SIFT	Fathm	Overall prediction
p.Tyr115Cys (c.344 A>G)	EGF1	Disease causing. score: 194	Probably damaging Score:1	Deleterious Score: 0	Damaging Score: −8.87	Damaging effect
p.Cys155Ser (c.464G>C)	EGF2	Disease causing. score: 112	Probably damaging Score:1	Deleterious Score: 0	Damaging Score: −15.72	Damaging effect
p.Arg75stop (c.223C>T)	GLA	Disease causing. score: 6	NA	NA	NA	Damaging effect
p.Glu63Metfs*7^a^ (c.187_188del)	GLA	Disease causing	NA	NA	NA	Damaging effect
p.Gly439Alafs*44^a^ (c.1314_1314delA)	Serine protease	Disease causing	NA	NA	NA	Damaging effect

^a^ Pathogenicity of frameshift mutation can only be predicted by the “Mutation taster” program.

^b^ Amino acids numbering followed HGVS nomenclature as Met is the first amino acid of premature FIX protein.

The c.1314_1314delA variant in exon 8 elongates the Factor IX protein by 20 amino acids, as predicted by Mutation Taster. This mutation can be explained by changing the original stop codon at 462 to the new position at 482 (p.Gly439Alafs*44) (see Figure S4 in Supplementary materials).

Molecular genetic analysis of carriers identified five mothers of patients as heterozygote carriers. The younger sister (HB‐012) of the severe patient (HB‐018) was found to be a non‐carrier. The results of the carrier detection test are shown in Table 4 and Supplementary Table S2. Relevant chromatogram figures of Sequence alignment of carriers have been shown in Figures 1 and 2 and Figures S1–S3. Sequence analysis was not performed on six female relatives (two mothers, three aunts, and one younger sister) from Family 2 and Family 4 because their male relatives (HB‐056 and HB‐059) did not have mutations in the entire *F9*.

**TABLE 4:** The bleeding tendency in carrier status.

Relative female ID	Carrying variants	Carrier status	MP (days)	PB	EB	PPH	RE	Usage of HA	ChLD
HB‐012	Wild type	Nc	3 to 5	No	Yes	No	No	No	No
HB‐072	c.464G>C	C	3	No	No	No	No	No	No
HB‐075	c.223C>T	C	7	No	No	No	No	No	No
HB‐071	c.187_188del	C	3 to 5	No	No	No	No	No	No
HB‐088	c.223C>T	C	4 to 6	No	No	No	No	No	No
HB‐065	NV in entire *F9*	ND	3 to 5	Yes	Yes	Yes	No	Yes	Yes
HB‐067		ND	5 <	No	No	No	No	No	No
HB‐068		ND	5 <	No	No	No	Yes	No	No
HB‐081	NV in entire *F9*	ND	3–5	Yes	Yes	Yes	No	No	No
HB‐082^a^		ND	NA	No	No	NA	NA	NA	No
HB‐108	c.1314_1314del	C	7	No	Yes	No	Yes	No	No

*Note*: PB more than 3 h after surgery or trauma (appendectomy, dental surgery, tonsillectomy, and others).

Abbreviations: C, carrier; ChLD, chronic liver disease; EB, easy bruises; HA, hemostatic agent; MP, menstrual period; NC, Non‐carrier; ND, not determined; NV, no variants; PB, prolonged bleeding; PPH, postpartum hemorrhage; RE, recurrent epistaxis.

^a^ This participant is 11 years old, and some questions did not applicable.

A total of 11 female relatives filled out the questionnaire form except HB‐066. Participant HB‐066 declined to fill out the questionnaire survey. The questionnaire survey revealed that indicative symptoms of being symptomatic carriers were not observed in four carriers (HB‐071, HB‐072, HB‐75, and HB‐088) identified through genetic testing. In contrast, the mother (HB‐065) of the mild patient (HB‐056) without mutation had a history of nearly all the symptoms of being a carrier (see Table 4). Additionally, this participant had chronic liver disease (chronic B hepatitis and infantile jaundice), potentially resulting in decreased clotting factors such as fibrinogen, Factor II, VII, and IX that are synthesized in the liver. The mother (HB‐081) with unknown carriership status has three significant symptoms, including prolonged bleeding, easy bruises, and postpartum hemorrhage. The mother (HB‐108) of the severe patient (HB‐110) had three significant symptoms such as a long menstrual period, easy bruises, and recurrent epistaxis of carriers. These findings suggest that the p.Gly439Alafs*44 (c.1314_1314delA) mutation might contribute to the symptoms of hemophilia carriers and the deficit of FIX activity.

## 4 DISCUSSION

This study aimed to initialize the molecular genetic analysis of hemophilia B and carrier detection in Mongolia to enhance genetic counseling. The study was conducted between 2014 and 2022 using a hospital‐based case study design. The study included cases previously diagnosed and receiving clotting factor replacement therapy. During the study period, the number of enrolled patients with HB accounted for a quarter of the total patients (*n* = 31). No cases with moderate HB were included in the study, which is likely attributed to the limited sample size. A total of five different variants were found in six out of eight patients. Two mild cases did not have a variant in the entire *F9*. Two unrelated patients with severe HB had the same recurrent nonsense mutation c.223C>T (p.Arg75*). According to the literature review, this mutation is more prevalent and, moreover, strongly linked to undetectable levels of Factor IX or a severe clinical presentation in individuals with hemophilia B across diverse ethnic populations (Belvini et al., 2005; Ivaskevicius et al., 2001; Knobloch et al., 1993; Kulkarni et al., 2021; Kwon et al., 2008; Li et al., 2014; Mårtensson et al., 2016; Parrado Jara et al., 2020; Yu et al., 2012). Thus, we cannot conclude that the above two patients have a common ancestor. Two missense mutations, p.Tyr115Cys (c.344A>G) and p.Cys155Ser (c.464G>C), were found in two severe patients with HB, respectively. The genotype and phenotype correlation of the above mutations is concordant with previous reports (Belvini et al., 2005; Knobloch et al., 1993; Salviato et al., 2019; Wulff et al., 1995; Yu et al., 2012). We found a novel p.Glu63Metfs*7 frameshift mutation (c.187_188del) in the patient with severe HB (HB‐080). The bioinformatic tool (Mutation taster) shows that the synthesis of several other domains, including EGF1 (amino acids: 93–130), EGF2 (amino acids: 131–173), linker (amino acids: 174–191), activation peptide (amino acids: 192–226), and serine protease (amino acids: 227–461), will be disrupted. The mutation will lead to the loss of various functions exhibited by the normal Factor IX. These functions include the activation of Factor IX protein (by the Gla domain), interaction with FVIIIa (by EGF1), binding to phospholipids and FVIIIa (by EGF2), and acquisition of full enzymatic activity (by the serine protease domain). A similar mutation was reported in the study of Belvini et al. (2005), p.Arg16fs*56 in severe patients. We found another novel frameshift p.Gly439Alafs*44(c.1314_1314delA) mutation in the patient with severe HB (HB‐110). The bioinformatics tool has revealed that this mutation leads to an elongation of the FIX protein by an extra 20 amino acids, affecting the serine protease domain. The serine protease domain plays a crucial role in catalyzing the cleavage of Factor X into Factor Xa and interacts with Factor VIIIa (Chavali et al., 2011). We postulate that elongation of the serine protease domain could potentially lead to a diminished affinity or interaction between Factor IX and Factor VIII. Certain variants in *F9* result in misfolding of the Factor IX protein by chaperones, which leads to impaired secretion of Factor IX (Enjolras et al., 2004). Hence, we postulate that improperly structured Factor IX with extended length may not be released in plasma from liver cells due to impaired post‐translational modification. Two patients with mild HB had no detectable variants in the *F9*. However, mutation screening in the promoter region, gene duplication analysis, and deep intron sequence should be investigated in these patients to find a genetic background of the disease phenotype. Because mutations in the promoter region were found in 1.3% (3/226) of the USA study (Belvini et al., 2005) and 1.27% (3/236) of the Italian study (Kwon et al., 2008), this mutation is also detected in either mild, moderate, and severe patients. Another study reported that mutations in the promoter region of the *F9* accounted for 7.8% (89) of 1113 unique mutations. We could not check for gene duplication of *F9* due to the lack of MLPA during the study period. However, gross duplication and deep intron variants have rarely been reported (Rallapalli et al., 2013). Our study also aimed to adopt a carrier detection test to improve the genetic counseling of HB in Mongolia. Carrier women who are heterozygous for a causative mutation in *F9* exhibit a diverse range of FIX levels. Those carriers with less than 40% of the normal FIX levels display symptoms resembling mild HB patients, such as excessive bleeding following invasive procedures. However, it depends on the mutation type or X‐chromosome inactivation (Miller, 2021). Neither FIX level nor pedigree analysis would be reliable for detecting the possible carrier. Thus, we mainly recruited the patient's mother in this study to validate the molecular genetic tests for carrier detection. The molecular genetic test results show that five mothers of five severe patients were heterozygote carriers. One severe patient's mother was not available during the study period. This case's younger sister (HB‐012) was a non‐carrier in this family. Almost all genetically identified carriers did not exhibit typical carrier symptoms. However, the phenotype and genotype concordance was observed only in the carrier mother (HB‐108, Family‐8), which was heterozygous for the c.1314_1314delA variant (Table 4). The screening of familial mutation among female relatives of childbearing age is beneficial for prenatal diagnosis of HB or risk assessment of postpartum hemorrhage. In the next study, other female relatives of the participating patients will undergo screening for the family‐specific mutation at their request. Identified mutations in this study might be used for prenatal diagnosis if female relatives request it. Almost all adult relative females of hemophilia patients want to know their carrier status for family planning. Mongolia is a developing lower‐middle‐income country with a small population, and half of the population lives in the capital city, Ulaanbaatar. Furthermore, we plan to set up molecular diagnostic tools in the clinical laboratory of Hemophilia Treatment Centers. The small sample size in this study depends on half of the patients with HB living in the countryside area, which is a long distance from Ulaanbaatar. Usage of FIX per capita in Mongolia is 5–13 times lower than that of high‐income countries with optimal treatment, such as Korea, Japan, Singapore, and Australia (Annual Global Survey 2021, WFH). Nevertheless, factor replacement treatment of HB in Mongolia is still suboptimal. Thus, carrier detection tests will be the most important key point of hemophilia care settings in Mongolia to prevent bleeding risks or delivery complications.

## 5 STUDY LIMITATIONS

Potential limitations should be noted in this report. Mutation identification in the promotor region gene duplication was not tested in this study. However, these mutations are rarely registered in the mutation database. We did not check for the hemophilia B Leiden mutation in two mild patients with HB. This mutation is the most frequent in the promotor region of *F9*. This mutation raises the FIX level after puberty, which is dependent on the androgen level, which induces the transcription factor of *F9* (Shen et al., 2022). A severe phenotype could become mild or moderate after puberty in patients with this mutation. In a literature review, about 40% (32) of 79 patients with the Leiden mutation show elevated FIX levels after puberty (Rallapalli et al., 2013; Shen et al., 2022). To understand the pathogenicity of two novel variants (c.187_188del and c.1314_1314delA) in this study, mRNA analysis of *F9* or western blotting of FIX protein could provide insights into mutation effects in our future studies.

## 6 CONCLUSION

The study marks the first molecular analysis of hemophilia B in Mongolia, encompassing carrier testing. Variants in the entire *F9* were found in six out of eight HB patients. Novel frameshift mutations, c.187_188del and c.1314_1314delA, were found to cause severe phenotypes. Symptoms in carrier women were notably infrequent among genetically identified heterozygous carriers. These results underscore the significance of molecular genetic tests of carrier detection in Mongolia's healthcare setting.

## AUTHOR CONTRIBUTIONS

Study conception and design: Munkhtsetseg B, Purevdorj I, Purevdorj M; sample collection: Ochbadrakh B and Enkh‐Amar A; draft revision: Munkhtsetseg B, Sarantuya J, Erkhembulgan P, Sodnomtsogt L; clinical data collection: Erdenebayar N and Enkh‐Amar A; supervisor on laboratory test: Ki‐Young Y; laboratory test, data analysis and interpretation of results, draft manuscript preparation: Purevdorj M. All authors reviewed the results and approved the final version of the manuscript.

## FUNDING INFORMATION

The study was supported by a research grant [Grant number: L2766‐Mon/No. 55] from the Mongolian Foundation for Science and Technology of The Ministry of Education and Culture Science of Mongolia.

## CONFLICT OF INTEREST STATEMENT

The authors declare that there is no conflict of interest regarding the publication of this paper. The authors stated that they had no interests, which might be perceived as posing a conflict or bias.

## ETHICS STATEMENT

A research Bioethical issue was approved by the Research Ethical Committee of the Mongolian National University of Medical Science (Approval number: 12–13/11). The authors confirm their contributions to the paper as follows: **Study conception and design:** Munkhtsetseg B, Purevdorj I, Purevdorj M; **sample collection:** Ochbadrakh B and Enkh‐Amar A; **draft revision:** Munkhtsetseg B, Sarantuya J, Erkhembulgan P, Sodnomtsogt L; **clinical data collection:** Erdenebayar N and Enkh‐Amar A; **supervisor on laboratory test:** Ki‐Young Y; **laboratory test, data analysis and interpretation of results, draft manuscript preparation:** Purevdorj M. All authors reviewed the results and approved the final version of the manuscript.

## PATIENT CONSENT STATEMENT

According to the Declaration of Helsinki, written informed consent was introduced to each of participating people and signed informed consent was obtained from participated person.

## Supporting information


Table S1:
Table S2:
Figure S1:
Figure S2:
Figure S3:
Figure S4:

## Data Availability

The data that supports the findings of this review and analysis are available in the supplementary material of this article. Sequence raw data of affected exons of two novel mutations will be available via Google Drive with the following link (https://drive.google.com/drive/folders/1FFFenCvJL95siWTb9oMcEn9XeEy7Ig3I?usp=sharing).
