# Supplementary material for: The genetic analysis of eight families with hemophilia B in Mongolia: Identification of two novel mutation
Source: Mol Genet Genomic Med. 2024 Sep 13;12(9):e2495. doi: 10.1002/mgg3.2495 (PMC11393770; doi:10.1002/mgg3.2495)
Supplement: Supplementary file 1 — Table S1: Table S2: Figure S1: Figure S2: Figure S3: Figure S4: [file MGG3-12-e2495-s001.docx]

**Supplementary**

**Supplementary tables:**

Table S1. Primer sequence for Exon PCR and Sanger sequence

| **Exons** | **Direction** | **Primer sequence**^†^ | **PCR Product length (bp)** |
| --- | --- | --- | --- |
| Exon 1 | Forward | 5'-CCCATTGAGGGAGATGGAC | 300 |
|  | Reverse | 5'-ATTGCTGTCAAATCATGTAATCA |  |
| Exon 2-3 | Forward | 5'-GCTCCATGCCCTAAAGAGAA | 565 |
|  | Reverse | 5'-CCCACATAATTCTCATATGTTTCA |  |
| Exon 4 | Forward | 5'-TGGCTTCCAGGTCAGTAGTTT | 300 |
|  | Reverse | 5'-TTCAACTTGTTTCAGAGGGAAA |  |
| Exon 5 | Forward | 5'-GCCCCCAATGTATATTTGACC | 300 |
|  | Reverse | 5'-TGTAGGTTTGTTAAAATGCTGAAG |  |
| Exon 6 | Forward | 5'-TGGGCCTCAATCTCAATTTT | 391 |
|  | Reverse | 5'-TCACATCCCAATAGGTCTGTCT |  |
| Exon 7 | Forward | 5'-TGCAAAGCTCACATTTCCAG | 381 |
|  | Reverse | 5'-CCTTCTGCCTTTAGCCCAAT |  |
| Exon 8 | Forward | 5'-TTGCCAATTAGGTCAGTGGTC | 778 |
|  | Reverse | 5'-TCCCCTGTAAAGAGAAAAAGCA |  |
|  | Forward | 5'-TCCTCAAATTTGGATCTGGC |  |

^†^-primers were purified with HPLC by the manufacturer company.

Table S2. The results of carrier detection in female relatives.

| Family number | Patient ID | Severity | Identified variants  (NM_000133.3). | Kinship female/ ID | Carrier status^†^ |
| --- | --- | --- | --- | --- | --- |
| Family-1 | HB-018 | Severe | c.344 A>G | Younger sister/HB-012 | Non-carrier |
| Family-3 | HB-057 | Severe | c.464G>C | Mother/HB-072 | Carrier |
| Family-5 | HB-060 | Severe | c.223C>T | Mother/HB-075 | Carrier |
| Family-6 | HB-080 | Severe | c.181_182delGA | Mother/HB-071 | Carrier |
| Family-7 | HB-085 | Severe | c.223C>T | Mother/HB-088 | Carrier |
| Family-8 | HB-110 | Severe | c.1314_1314delA | Mother/HB-108 | Carrier |

^†^-Carrier status was determined by the molecular genetic test based on the identified variants in male relatives.

**
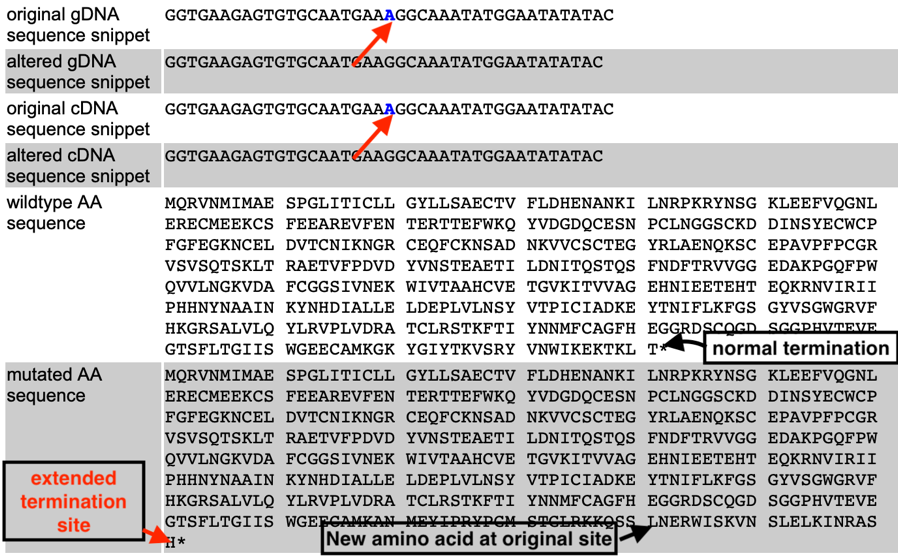

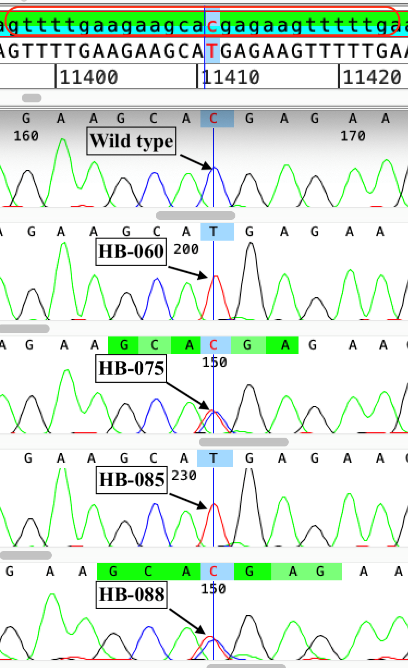

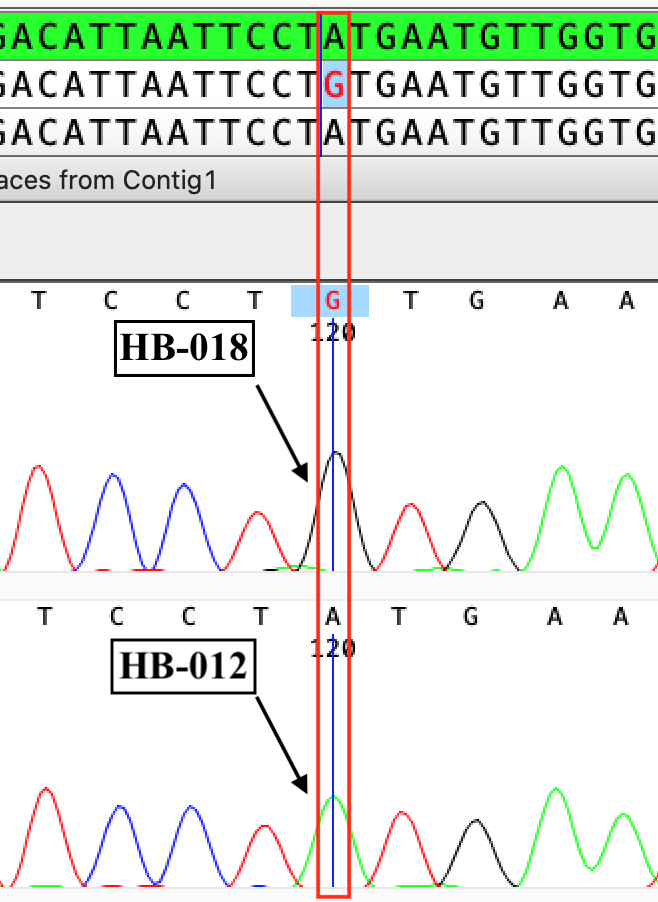

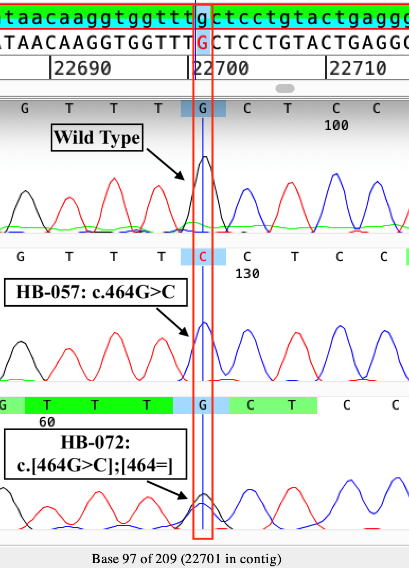
Supplementary** **figures:**

**Figure S2. Sequence chromatogram of c.344A>G mutation.** The reference sequence (NG_007994.1) has been highlighted in green. The c. 344A> G mutation was found in a patient with HB-018 ID. His younger sister (HB-012) was identified as a wild-type homozygote. The target position is shown in the red line.

**Figure S1. Sequence chromatogram of c.464G>C mutation.** The reference sequence (NG_007994.1) has been highlighted in green. The c.464G>C mutation was found in a severe Patient (ID: HB-057); his mother (ID: HB-072) is a heterozygote carrier for this mutation. The wild type is shown at the top.

**Figure S4. Comparison of amino acid sequence between the wild type and mutated Factor IX due to c.1314_1314delA mutation (via Mutation taster software, ref sequence used in FIX protein (**NP_000124.1**).** Factor IX protein is terminated by “Threonine” amino acid at position 461. c.1314_1314delA mutation leads to frameshift mutation, which results in the last amino acid, “Threonine” has been changed by “Leucine” at position 461. The protein length is extended until 482 amino acids and is terminated by Histidine amino acid. The overall length of Factor IX protein is extended by 20 amino acids; the most affected domain by this frameshift mutation is the serine protease.

**Figure S3. Sequence chromatogram of c.223C>T mutation.** The reference sequence (NG_007994.1) has been highlighted in green. The mutation was found in two unrelated patients (HB-060 and HB-085). HB-075 is the mother of patient HB-060, and HB-088 is the mother of patient HB-085. The black arrow shows the mutated point.
